# Nutrition-Related Mobile Apps in the Spanish App Stores: Quality and Content Analysis

**DOI:** 10.2196/52424

**Published:** 2024-10-04

**Authors:** Cesar I Fernandez-Lazaro, Gema Santamaría, Annika Fernandez Milano, Maria I Martin-Vergel, Diego Fernandez-Lazaro

**Affiliations:** 1 Department of Preventive Medicine and Public Health, School of Medicine, University of Navarra, Pamplona, Spain; 2 IdiSNA, Navarra Institute for Health Research, Pamplona, Spain; 3 Departamento de Anatomía y Radiología, Facultad de Ciencias de la Salud, Campus de Soria, Universidad de Valladolid, Soria, Spain; 4 IQVIA Commercial GmbH & Co OHG, Frankfurt am Main, Germany; 5 Departamento de Ciencias Farmacéuticas y de la Salud, Facultad de Farmacia, Universidad CEU San Pablo, Madrid, Spain; 6 Grupo de Investigación Reconocido “Neurobiología,” Facultad de Medicina, Universidad de Valladolid, Valladolid, Spain; 7 Departamento de Biología Celular, Genética, Histología y Farmacología, Facultad de Ciencias de la Salud, Campus de Soria, Universidad de Valladolid, Soria, Spain

**Keywords:** mobile apps, mHealth, mobile health, app, nutritional, nutrition, dietary, eating, diet, food, lifestyle, Spain, Spanish, chronic diseases, chronic, review, quality, MARS, Mobile App Rating Scale, uMARS, user version of the Mobile App Rating Scale, assessment, mobile phone

## Abstract

**Background:**

Mobile apps represent accessible and cost-effective tools to improve nutrition and prevent chronic diseases. However, most of these apps have been characterized as having limited functionality, raising concerns about their effectiveness, acceptability, and efficacy.

**Objective:**

The aims of the study were to assess the quality of popular nutrition-related app platforms in Spain and to describe their characteristics and functionalities.

**Methods:**

We screened apps providing information on dietary advice, food advice, and nutritional content in the Apple App Store and Google Play Store in Spain from March 2 to March 16, 2024. Apps with a star rating of ≥4 (of 5 stars), those available in Spanish, those that were free of charge, those last updated after January 2022, those with >500 reviews, and those with >500,000 downloads were included. The quality of apps was assessed using the user version of the Mobile App Rating Scale (uMARS). General characteristics and nutritional, health, and market-related functionalities of the nutrition-related apps were described. Correlations among total and uMARS sections, star ratings, and number of reviews and downloads were evaluated.

**Results:**

Among the 1460 apps identified in the search, 42 apps met the criteria. The majority of these (n=20, 48%) aimed at recording and analyzing food intake, followed by those providing nutritional plans or diets (n=9, 21%), advising on healthy habits (n=7, 17%), and offering recipes (n=6, 14%). The most prevalent nutritional functionalities offered were recording and monitoring body measurements (n=30, 71%), food tracking (n=26, 62%), and dietary analysis (n=25, 60%), whereas nutrition education was less common (n=16, 38%). Among market-related functionalities, advertisements were the most common among the study apps (n=30, 71%), followed by the option of sharing on social media (n=29, 69%) and customizable reminders (n=26, 62%). Sharing the recorded information in the app with health professionals was infrequent (n=1, 2%). The mean (SD) total uMARS score (maximum 5 points) was 3.78 (0.35), while the mean (SD) uMARS scores for functionality, aesthetics, engagement, and information were 4.21 (0.38), 3.94 (0.54), 3.51 (0.46), and 3.48 (0.44), respectively. Lower mean scores were observed for the subjective quality (mean 2.65, SD 0.56) and perceived impact (mean 3.06, SD 0.67). Moderate to strong positive significant correlations were mostly observed between total uMARS and section-specific uMARS scores, while the correlations between the uMARS section scores were mostly moderate positive. Total uMARS scores were very weakly correlated with user rating, number of reviews, and number of downloads.

**Conclusions:**

The quality of popular nutrition-related app platforms in Spain was acceptable, with observed remarkable differences between sections. The majority of the apps were appealing due to their user-friendly interfaces. Only a few apps, however, provided dietary structure analysis or nutritional education. Further research is needed to assess the long-term impact of these apps on users.

## Introduction

Noncommunicable diseases—commonly known as chronic diseases—are responsible for more than 60% of the global disease burden, representing a public health concern [1]. Their high prevalence and mortality rates constitute a threat to individuals, families, health care systems, and governments due to their health and economic impacts [2,3]. Mounting and robust scientific evidence suggests that nutrition, a potentially modifiable lifestyle factor, is linked to chronic diseases, opening up new opportunities for their prevention [4,5]. For instance, a recent umbrella review of meta-analyses, which included 116 primary prospective cohort studies, revealed inverse associations between healthy dietary patterns and risk of chronic diseases, including type 2 diabetes, certain types of cancer, and cardiovascular disease, among others. Conversely, positive associations were found between unhealthy dietary patterns and the risk of chronic diseases [6]. Additionally, extensive research on the Mediterranean diet, one of the most studied and well-known dietary patterns globally, has consistently demonstrated its positive impact on cardiovascular health. This includes a decreased risk in developing lifestyle diseases, such as obesity, hypertension, metabolic syndrome, and dyslipidemia, as well as being linked to lower rates of diabetes, glycemic control, and an age-related cognitive decline [7,8].

In the search for accessible and cost-effective interventions to improve nutrition and prevent chronic diseases, mobile health (mHealth) apps have emerged as an extension of traditional approaches [9]. mHealth apps may provide continuous monitoring, health care provider communication and support, reminders, patient education, and patient involvement strategies that facilitate chronic disease management [10,11]. Moreover, mHealth apps have proven to be effective in achieving healthier lifestyles through behavioral change by helping to increase physical activity levels and improve dietary patterns [12-14]. These relatively new digital health methods stand out for their low cost, innovative functionalities, and high usability [15]. Moreover, the use of these apps has remarkably increased in recent years [16-18]. Currently, 6.9 billion users—86.1% of the world population—own a smartphone [19] with access to more than 350,000 apps in top app stores in 2021 (with approximately 90,000 new mHealth apps added in 2020) [15]. Among these apps, approximately 11% were categorized as diet and nutrition-related apps.

The thousands of mHealth apps available in top stores enable smartphone users to choose the app that best meets their needs. However, over 50% of mHealth apps have limited functionality [20], and there are several concerns about their effectiveness, acceptability, and efficacy [21,22], indicating room for improvement [23]. Moreover, reports suggest that most users stop using mHealth apps after installation or a few interactions [24,25] due to a lack of desired features or the apps not being easy to use [26].

Spain is among the European countries with the highest smartphone penetration rates, with over 47 million people (97% of the population) owning a smartphone. The use of mHealth apps is also continuously increasing, particularly the nutrition-related apps that account for 34% of the mHealth apps market [27,28]. No studies, however, have been conducted to evaluate the quality and functionality of nutrition apps in the Spanish app stores. Therefore, the aims of this study were to evaluate the quality of popular nutrition-related app platforms in Spain and describe the different characteristics and functionalities of these apps.

## Methods

### Search Strategy

The iOS (Spanish Apple App Store) and Android (Spanish Google Play Store) platforms were used to search for nutrition-related apps from March 2 to March 16, 2024. The search on the Android platform was conducted via laptop, using the Google Chrome browser in an incognito window while logged out of the Google account to prevent any influence of user preferences. The search on the iOS platform was conducted on a mobile device, an iPhone 11 Pro. Of note, the displayed apps may differ from those in other countries due to the search having been carried out in Spanish app stores. The following Spanish search terms were used: “Nutrición” (nutrition), “Dieta” (diet), and “Dieta saludable” (healthy diet). All results for each keyword on both platforms were reviewed.

### Screening and Selection Criteria

The selection process of the apps was conducted following the PRISMA (Preferred Reporting Items for Systematic Reviews and Meta-Analyses) [29]. All apps were initially independently screened and analyzed by 2 study researchers (GS and MIM-V) based on the description and screenshots on their download page, and disagreements between them were resolved by a third reviewer (CIF-L). We based the selection of apps on the following criteria: (1) a minimum rating of 4 of 5 stars; (2) available in the Spanish language; (3) freemium (free of charge); (4) last update after January 2022; (5) more than 500 reviews; (6) more than 500,000 downloads; and (7) dietary advice (eg, diet plan or meal planning), food advice (eg, cooking process or characteristics of food), or nutritional information of food or drinks (eg, nutrient content or recommended intake). The following exclusion criteria were applied: (1) apps initially free of charge but with a monthly subscription later on, (2) apps used as a food diary without dietary analysis, (3) apps requiring external equipment (scales and smartwatches) for use, (4) apps designed for use by health care professionals, (5) restaurant apps for making reservations or displaying menus, (6) apps for taking photos or videos of food, (7) educational food-related apps for children, (8) apps that allow offline payments for health services, (9) apps that exclusively monitor weight and physical activity or display recipes (without including nutritional information), and (10) apps targeted at specific populations such as pregnant women or individuals diagnosed with diabetes.

After identifying the nutrition-related apps that met all the inclusion criteria and none of the exclusion criteria, duplicates within and between the platforms were removed before each researcher downloaded and explored the remaining apps. Following a more in-depth analysis, apps were examined by the same study researchers and selected for further analysis based on the abovementioned criteria.

### Data Extraction

The following general characteristics were extracted from the nutrition-related apps that met these criteria: number of downloads, number of reviews, number of stars, date of last update, and a brief description of the main goal of the apps. Since the Apple App Store does not provide the number of downloads, this information and the rest of the characteristics related to the apps were extracted from the Google Play Store when the apps were available on both platforms. In addition, the nutritional, health, and market-related functionalities of these apps were extracted by the study researchers after a literature review and discussions with experts and were recorded in a Microsoft Excel spreadsheet (Microsoft Corp).

### Quality Assessment Using the User Version of the Mobile App Rating Scale

The user version of the Mobile App Rating Scale (uMARS) was used to evaluate the quality of the apps [30]. The uMARS is an adaptation of the Mobile App Rating Scale (MARS) developed for end users, while the MARS is used by professionals who require training and expertise in mHealth in order to perform the assessment [31]. The uMARS is composed of 4 objective sections, each containing multiple items: engagement, functionality, aesthetics, and information. The engagement section (5 items) assesses how entertaining, interesting, customizable, and interactive the app is and whether it fits the target group. The functionality section (4 items) tests the app’s performance, ease of use, navigation, and gestural design. The aesthetics section (3 items) evaluates the layout, graphic design, and overall visual appeal. Finally, the information section (4 items) focuses on the quality and quantity of the information, the visual information, and the credibility of the sources. All items are assessed on a 5-point scale (1=inadequate, 2=poor, 3=acceptable, 4=good, and 5=excellent). In addition, the uMARS includes the sections subjective quality (4 items) and perceived impact (6 items), which evaluate whether the app may increase awareness, change attitudes, and promote changes toward healthier behaviors. The same 2 study researchers (GS and MIM-V) independently assessed the quality of the nutrition-related apps using the uMARS. The researchers evaluated the apps from the perspective of users rather than experts.

### Statistical Analysis

Descriptive statistics, including frequency and proportions, were used to summarize the general characteristics and the nutritional, health, and market-related functionalities of the selected apps. The overall and section-specific uMARS scores, along with the uMARS scores by the main aim of the apps, were described by their maxima, minima, means, medians, and IQRs and were graphically represented by boxplots. Pearson and Spearman correlation coefficients for normally and nonnormally distributed data, respectively, were determined between app characteristics and total and section-specific uMARS scores. The distribution of the data was checked with the Shapiro-Wilk test. All analyses were performed with Stata (version 16.0; StataCorp LLC), with a 2-tailed level of statistical significance set at *P*≤.05.

### Ethical Considerations

The research conducted in this study did not require the participation of human participants, and no personal data were referenced or collected. The data used in the study were obtained solely from publicly available sources on app stores, and as a result, ethics approval was not needed. This rationale aligns with the institutional policies of the research location [32].

## Results

### Search Results

**Figure 1.**
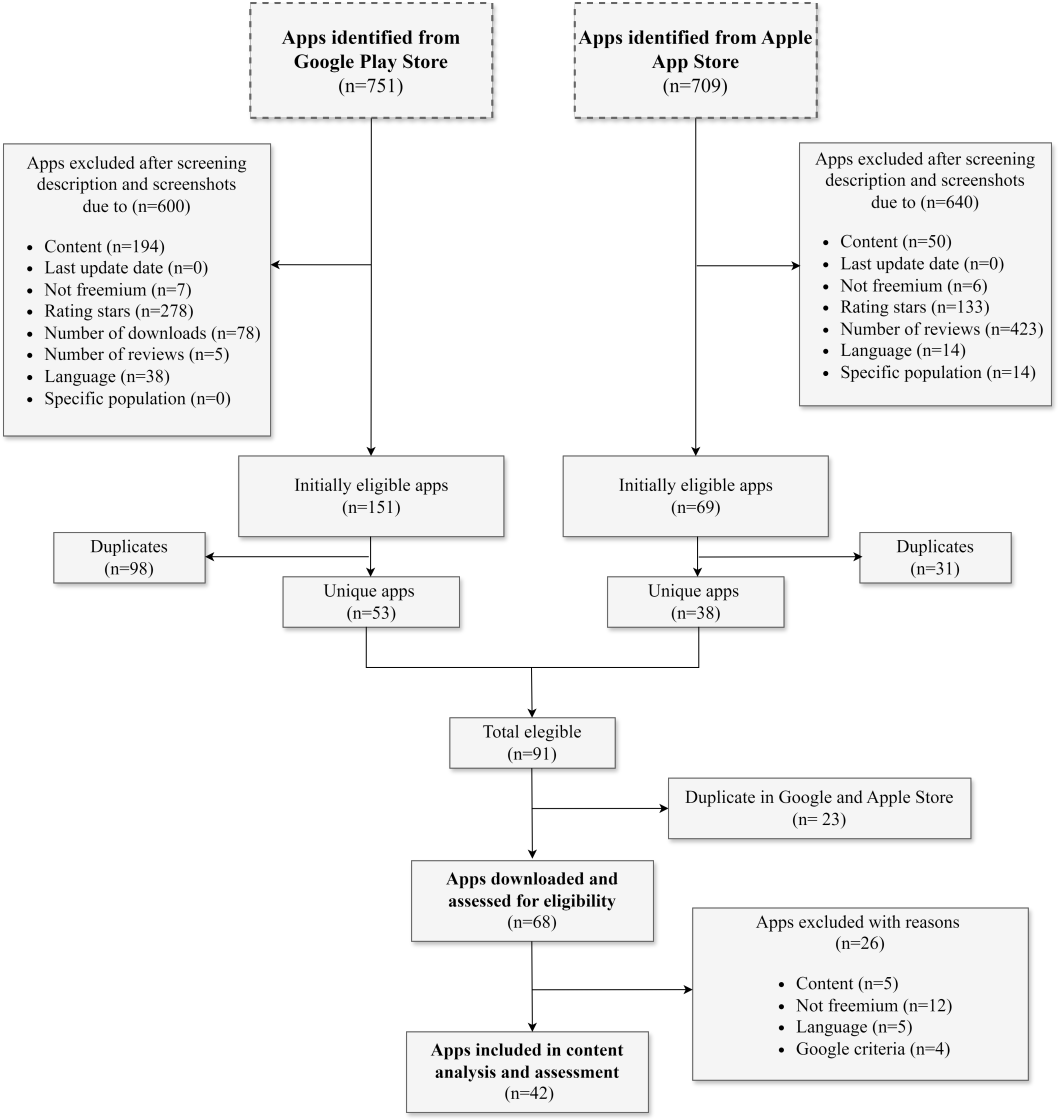
Flowchart of the selection of nutrition-related apps for the in-depth analysis and quality assessment.

Our search yielded a total of 1460 (n=751 from Google Play Store and n=709 from Apple App Store) apps (Figure 1). Of these, 1240 were excluded for not meeting the inclusion criteria (Google Play Store [n=600]: content: n=194, not freemium: n=7, rating stars: n=278, number of downloads: n=78, number of reviews: n=5, and language: n=38; and Apple App Store [n=640]: content: n=50, not freemium: n=6, rating stars: n=133, number of reviews: n=423, language: n=14, and targeted specific population, n=14). After removing duplicates on the same platform (Google Play Store: n=98 and Apple App Store: n=31) and between the Google Play Store and Apple App Store (n=23), the remaining 68 apps were downloaded and further assessed for eligibility. Of these, 26 apps were then excluded (content: n=5, not freemium: n=12, language: n=5, and Google criteria: n=4), resulting in a total of 42 nutrition-related apps that were included in the study for an in-depth analysis.

### General Characteristics of the Nutrition-Related Apps

The general characteristics of the 42 apps included in the study are displayed in Table 1. A large proportion of the apps (n=21, 50%) were exclusively available on the Android platform, and only a small proportion was exclusively available on the iOS platform (n=5, 12%). The remaining apps (n=16, 38%) were available on both platforms. A great proportion of the selected apps had a rating of 4.6 to 4.8 stars (n=18, 43%), at least 50,000 reviews (n=17, 41%), 1.0 to 4.9 million downloads (n=18, 43%), and offered a premium version with additional paid features (n=38, 91%). Furthermore, the primary aim of many apps was to record and analyze food intake (n=20, 48%), followed by providing nutritional plans or diets (n=9, 21%), advising on healthy habits (n=7, 17%), and offering recipes (n=6, 14%).

**Table 1.** General characteristics of the nutrition-related apps included in the study (N=42).

Characteristics	Values, n (%)
**Platform**	
Android	21 (50)
iOS	5 (12)
Android and iOS	16 (38)
**User star rating**	
4.0‐4.2	6 (14)
4.3‐4.5	16 (38)
4.6‐4.8	18 (43)
4.9‐5.0	2 (5)
**Number of reviews (in thousands)**	
<5.0	9 (21)
5.0‐14.9	10 (24)
15.0‐49.9	6 (14)
≥50.0	17 (41)
**Number of downloads (in millions)**	
Missing	5 (12)
<1.0	4 (10)
1.0‐4.9	18 (43)
5.0‐9.9	5 (12)
10.0‐49.9	7 (17)
≥50.0	3 (7)
**Premium version**	
Yes	38 (91)
No	4 (9)
**Main aim**	
Nutritional plan or diet	9 (21)
Recipes	6 (14)
Recording and analyzing food intake	20 (48)
Advice on healthy habits	7 (17)

### Nutritional and Health Functionalities

The nutritional and health functionalities of the apps included in the study are described in Table 2. More than half of the apps offered a search function for food and nutritional information (n=23, 55%), specifically on energy content (n=23, 55%) and macronutrients (n=21, 50%). A lower proportion of apps informed about fiber (n=9, 21%), micronutrients (n=6, 14%), and food additives (n=2, 5%). Around two-thirds of the apps included a function to record food intake (n=26, 62%), of which quantification by weight (n=21, 50%) and portion size (n=19, 45%) were the most common. Recipes and dietary plans were provided by 48% (n=20) of the apps, and around one-third of these were customizable to the user’s preferences, intolerances, or lifestyle (n=14, 33%). The majority of the apps also offered the recording and monitoring of body measurements (n=30, 71%), such as weight (n=30, 71%) and BMI (n=14, 33%). In addition to nutritional functionalities, a considerable number of apps provided health functionalities related to physical activity (n=25, 60%), with the most common functionalities being type (n=23, 55%) and duration of physical activity (n=22, 52%). Among the apps that offered dietary analysis and suggestions (n=25, 60%), most provided recommendations on water and calorie intake (n=24, 57%) and energy analyses (n=23, 55%), followed by dietary structure analyses (n=20, 48%). The fewest apps provided nutritional education (n=16, 38%).

**Table 2.** Nutritional and health functionalities of the nutrition-related apps included in the study (N=42).

Functionalities	Values, n (%)
**Searching food and providing nutritional information**	23 (55)
Energy	23 (55)
Macronutrients	21 (50)
Micronutrients	6 (14)
Food additives	2 (5)
Fiber	9 (21)
**Recording food intake**	26 (62)
Quantified by weight	21 (50)
Quantified by portion	19 (45)
Favorite foods	17 (41)
Water consumption	18 (43)
**Providing recipes and dietary plans**	20 (48)
Customizable according to preferences, intolerances, or lifestyle	14 (33)
With specific amount of food	12 (29)
Shopping list	12 (29)
**Recording and monitoring body measurements**	30 (71)
Weight	30 (71)
Waist circumference	11 (26)
BMI	14 (33)
**Recording physical activity**	25 (60)
Type of physical activity	23 (55)
Duration of physical activity	22 (52)
Burned calories	20 (48)
Pedometer	7 (17)
**Dietary analysis and suggestions**	25 (60)
**Energy analysis**	23 (55)
Total energy	23 (55)
Energy balance	12 (29)
Energy ratio of meals	5 (12)
**Dietary structure analysis**	20 (48)
Food groups	1 (2)
Distribution of macronutrients	19 (45)
Monitoring of micronutrient intake and advising on required minimum	1 (2)
Target weight and BMI	11 (26)
Water and calorie intake recommendations	24 (57)
**Nutritional education**	16 (38)
Independent education module	14 (33)
Minimum micronutrient requirements, their function, and sources	3 (7)

### Market-Related Functionalities

The market-related functionalities of the included apps are described in Table 3. Advertisements were the most common market-related functionality among the selected apps (n=30, 71%), followed by the option of sharing on social media (n=29, 69%) and customizable reminders (n=26, 62%). A total of 43% (n=18) of the apps provided challenges and incentives such as points, badges, or rankings to enhance users’ motivation. A more novel function, sharing the information recorded in the app with health professionals, was only provided by 1 (2%) app, while communication with other users was available in 13 (31%) apps. A total of 21 (50%) apps included in the study offered a method to recognize foods through intelligent recognition technology; specifically, 17 (41%) apps offered barcode or QR code recognition, and 14 (33%) offered photo recognition.

**Table 3.** Market-related functionalities of the nutrition-related apps included in the study (N=42).

Functionalities		Values, n (%)
Communicating with other users		13 (31)
Sharing on social media		29 (69)
Receiving invitations to challenges and incentives		18 (43)
Receiving reminders (push notifications)		26 (62)
Receiving advertisement		30 (71)
Buying products		3 (7)
Sharing with health professionals		1 (2)
**Intelligent recognition technology**		21 (50)
	Barcode or QR code	17 (41)
	Photo	14 (33)

### Assessment of the Quality Content

**Figure 2.**
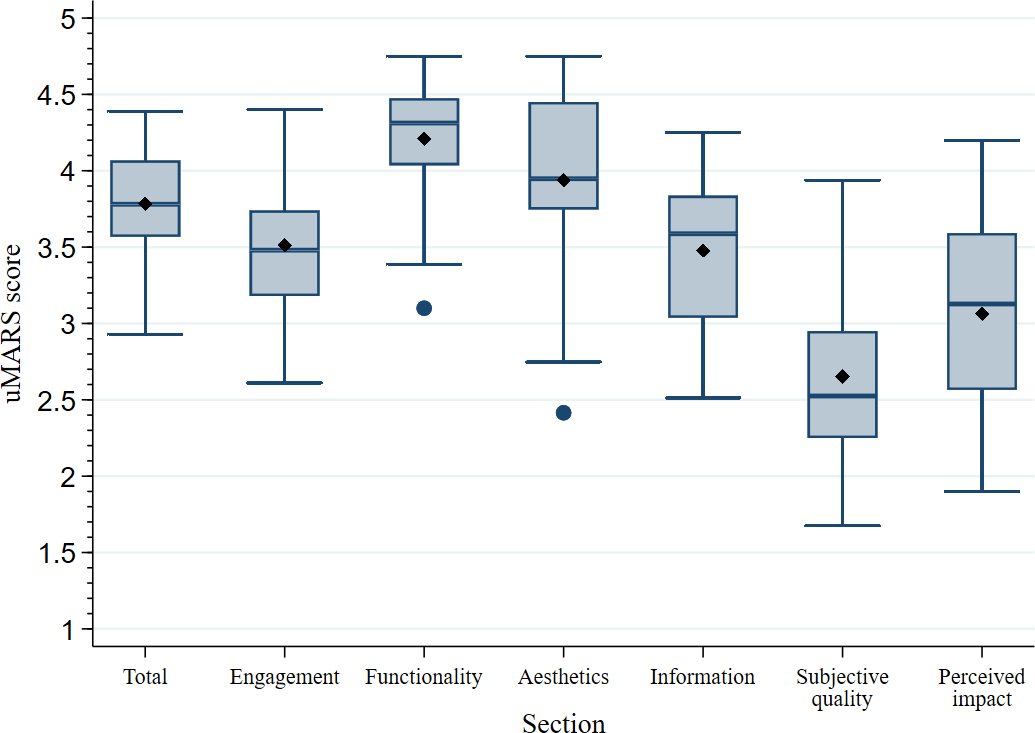
Quality assessment of the nutrition-related apps of the study (N=42). The box plots represent the mean total and section-specific scores of the user version of the Mobile App Rating Scale (uMARS) assessment scores. The line within the boxes represents the median score, the diamond within the boxes represents the mean score, the dot outside the boxes represents an outlier score, and the whiskers represent the maximum and minimum values (except in the presence of outliers).

The quality content of the selected nutrition-related apps was assessed using the uMARS scale. The results of the assessment are displayed in Figure 2. A total of 12 (29%) nutrition-related apps scored a mean higher than 4 of 5 points on the uMARS scale. The app with the highest mean total score was 4.39 (SD 0.32), and the lowest mean total score was 2.93 (SD 0.86). The mean total score for all the apps was 3.78 (SD 0.35), and the total median score was 3.78 (IQR 3.57-4.07). Regarding uMARS section-specific scores, functionality was the section with the highest scores, with a mean of 4.21 (SD 0.38), followed by aesthetics (mean 3.94, SD 0.54), engagement (mean 3.51, SD 0.46), and information (mean 3.48, SD 0.44). There was some variability in the scores for each section of the uMARS, with functionality scores ranging from 3.10 to 4.75, aesthetics scores from 2.42 to 4.75, engagement scores from 2.61 to 4.40, and information scores from 2.51 to 4.25.

The sections subjective quality and perceived impact of the uMARS were additionally assessed. The mean scores for the subjective quality and perceived impact sections were lower at 2.65 (SD 0.56) and 3.06 (SD 0.67), respectively, than those of the previously described app quality sections (engagement, functionality, aesthetics, and information). With the exception of aesthetics, a wider range of scores was observed for the perceived impact (1.90 to 4.20) and subjective quality (1.67 to 3.94). Details of the quality content assessment can be found in Multimedia Appendix 1.

**Figure 3.**
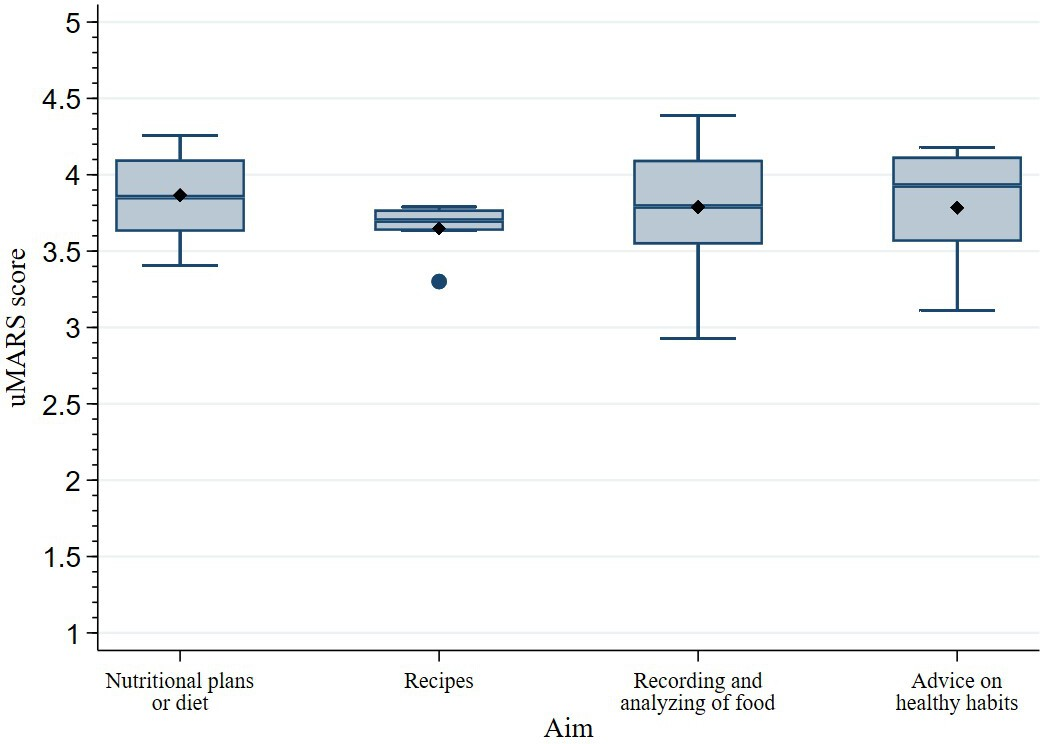
Quality assessment according to the aim of the nutrition-related apps of the study (N=42). The box plots represent the mean total score of the user version of the Mobile App Rating Scale (uMARS) assessment by the aim of the apps. The line within the boxes represents the median score, the diamond within the boxes represents the mean score, the dot outside the boxes represents an outlier score, and the whiskers represent the maximum and minimum values (except in the presence of outliers).

The results of the quality content assessment, according to the main aim of the selected nutrition-related apps, are displayed in Figure 3. The apps whose main aim was to provide nutritional plans or diets were the best rated, with a total mean score of 3.87 (SD 0.33). They were followed by the apps that recorded and analyzed food intake (mean 3.79, SD 0.39), the apps that mainly provided advice on healthy habits (mean 3.78, SD 0.37), and those that primarily offered recipes (mean 3.65, SD 0.18). App-specific scores can be found in Multimedia Appendix 2.

### Relationship Between App Characteristics, Total uMARS Scores, and Section-Specific uMARS Scores

Pearson and Spearman correlation coefficients between app characteristics and total and section-specific uMARS scores are shown in Table 4. Overall, strong positive significant correlations were observed between total uMARS and section-specific uMARS scores, while the correlations between the uMARS section scores were mostly moderate positive. Notably, total uMARS scores were weakly correlated with user rating, number of reviews, and number of downloads.

**Table 4.** Correlations between app characteristics and total and section-specific user version of the Mobile App Rating Scale (uMARS) scores of the nutrition-related apps included in the study (N=42).

Characteristics		uMARS total	User star rating	Number of reviews	Number of downloads^a^	uMARS section-specific				
						Engagement	Functionality	Aesthetics	Information	Subjective quality
**User star rating**										
	Value	0.256^b^								
	*P* value	.13								
**Number of reviews**										
	Value	0.196^b^	0.031^b^							
	*P* value	.25	.85							
**Number of downloads** ^a^										
	Value	0.135^b^	0.113^b^	0.860^b^						
	*P* value	.43	.51	<.001						
**uMARS engagement**										
	Value	0.791^c^	0.221^b^	0.191^b^	0.151^b^					
	*P* value	<.001	.19	.26	.37					
**uMARS functionality**										
	Value	0.767^b^	0.158^b^	0.116^b^	0.165^b^	0.353^b^				
	*P* value	<.001	.35	.50	.33	.03				
**uMARS aesthetics**										
	Value	0.743^b^	0.218^b^	0.324^b^	0.260^b^	0.444^b^	0.613^b^			
	*P* value	<.001	.19	.05	.12	.006	<.001			
**uMARS information**										
	Value	0.708^c^	0.123^b^	0.003^b^	0.098^b^	0.618^c^	0.451^b^	0.290^b^		
	*P* value	<.001	.47	.99	.57	<.001	.005	.08		
**uMARS subjective quality**										
	Value	0.757^c^	0.220^b^	0.143^b^	0.203^b^	0.788^c^	0.592^b^	0.346^b^	0.675^c^	
	*P* value	<.001	.19	.40	.23	<.001	<.001	.03	<.001	
**uMARS perceived impact**										
	Value	0.735^c^	0.147^b^	0.178^b^	0.138^b^	0.810^c^	0.497^b^	0.413^b^	0.778^c^	0.763^c^
	*P* value	<.001	.39	.29	.42	<.001	.002	.01	<.001	<.001

a Information on the number of downloads was missing for 5 apps.

b Correlation assessed using Spearman rank correlation.

c Correlation assessed using Pearson rank correlation.

## Discussion

### Principal Results

To the authors’ knowledge, this is the first study conducted with apps from the Spanish app stores (on the iOS and Android platforms) aimed at evaluating the quality of the most popular and freemium nutrition-related apps and at describing their characteristics and functionalities. The study search identified 42 apps that met the inclusion criteria, most of which were aimed at recording and analyzing food intake. The overall quality of the apps included in the study was acceptable; however, remarkable differences between the section-specific quality scores of the uMARS were revealed, with functionality having received the highest rating of the 4 uMARS sections.

The most common functions that the apps provided were recording of body measurements and food intake, searching for food and nutritional information, and conducting dietary analysis. Monitoring and search functions were also found to be the most common strategies in nutrition-related apps to elicit behavioral change, as indicated elsewhere [33,34]. Only a few apps included in this study offered nutritional education or recipes and dietary plan functionalities, for which a higher development burden could be the reason. The elaboration of these functions is costly and time-consuming, as it requires close collaboration with health care professionals [33].

### Comparison With Prior Work

The mean total uMARS score in our study (3.78, SD 0.35) was slightly higher compared to similar nutrition-related app studies conducted in China (3.5) [33] and Korea (2.9) [34]. Unlike our study, the Chinese study [33] included apps regardless of ratings and downloads, while the Korean study [34] used the MARS (opposed to the uMARS used in our study), which requires an assessment by experts rather than by users. Experts may be more inclined to evaluate the quality of the apps more rigorously. Thus, these methodological differences may explain the assessment disparities between the previous studies [33,34] and our study.

In our study, the information section of the uMARS scored the lowest among the 4 main sections, consistent with the study by Martinon et al [35], in which only one-third of the apps offered scientific evidence. This raises concerns about the validity of the information. The information conveyed through apps should be based on empirical evidence, and its accuracy should be rigorously verified, as misinformation can negatively affect users’ well-being and health goals. A specialized grading scale designed to evaluate nutritional content could be beneficial in assessing the accuracy of information more effectively [33]. Previous studies have reported overestimations [36] or underestimations [37] of energy intake, while others have found close alignment with validated reference methods [38,39]. The variability in energy intake and nutrient measurements across apps may be explained by the lack of alternative serving sizes offered by the apps [40] and the use of different databases based on distinct nutritional reference guides [36,38,39]. Moreover, self-reported methods of nutritional assessment are subject to a degree of measurement bias, such as underestimation and overestimation of dietary intake, which poses an additional challenge for accuracy calculations [41]. An image-based assessment of the food type and portion could counteract this bias [42]. Along with water and calorie intake recommendations and dietary structure analysis, energy analysis has emerged as one of the most common functionalities offered by dietary analysis apps in our study. In another study [43], energy analysis and calorie intake recommendations were even more prevalent among the apps examined. A possible explanation for this dominance could be the ease of implementing energy calculations within an app [33] and users’ interest in tracking energy intake to pursue weight loss goals, as self-monitoring is a crucial element of behavioral weight loss [44,45]. However, the assessment of food group composition or dietary patterns may provide a more comprehensive approach to disease prevention or treatment [46]. In our study and similar research, only a few of the included apps that provided dietary structure analysis actually assessed food group composition [33,39].

Despite the majority of the apps including reminders to inform users about unachieved objectives, and nearly half of the apps offering challenges and incentives, the engagement section scored the second lowest of the 4 main sections, similar to elsewhere [33]. Ongoing motivation is fundamental to continuously engage individuals in successfully achieving app goals and sustaining healthy habits in the long term [47,48]. The inclusion of customizable reminders [49], and in particular gamification, such as receiving digital rewards once a challenge has been successfully completed, may increase the user’s motivation through positive, playful experiences [40]. In addition, users seek social support through preferably close-knit communities and continuous guidance by health care professionals. Due to time constraints [47] and a lack of nutritional training [50], physicians often struggle to provide support to their patients regarding nutritional changes. Nutrition-related and other mHealth apps may facilitate the establishment of multidisciplinary health care teams that include dietitians, who can provide nutritional counseling and long-term motivational support to patients. The importance of involving dietitians in promoting dietary lifestyle changes has been studied elsewhere [51,52]. The app environment simplifies the collaboration of different professional groups within the health care system, which would not be feasible otherwise due to location and time constraints.

The functionality and aesthetics sections scored the highest and second highest, respectively. Previous studies have reported functionality as the top uMARS section [33,35,53,54]. When designing apps, it is fundamental to ensure that they work seamlessly and intuitively, as apps that are easy to navigate have been found to reduce usability barriers and motivate people who are less familiar with technology [55]. Regarding aesthetics, the inclusion of visual elements, such as pictures, food icons, or charts, has been previously described as enhancing the user experience [33,34]. For instance, Li et al [33] found that apps with higher aesthetic ratings performed better in overall uMARS scores than those with lower ratings.

The other 2 additional sections of the uMARS, namely, “subjective quality” and “perceived impact,” were rated the lowest, which is consistent with findings from other studies [34,35,54]. The low subjective score in relation to the overall uMARS score implies that despite good ratings for engagement, functionality, aesthetics, and information quality, the raters would not recommend the app [35].

### Limitations

The results of the study should be interpreted in light of the following limitations. First, this study only included nutrition-related apps in the Spanish language from the app stores in Spain, excluding poorly rated apps (<4 stars), those not free of charge, those with fewer than 500 reviews, and those with fewer than 500,000 downloads. Therefore, this may limit the generalizability of the findings to freemium and the most popular nutrition-related apps in the Spanish app store. Second, since the apps were evaluated from the perspective of users, the assessments could vary from those made by nutrition experts or a joint evaluation by both groups. Third, despite the uMARS [30] having been used in other nutrition-related assessment studies [33] and being considered a reliable measure of app quality with similar psychometric characteristics and results to the standard version [30,31], the use of questionnaires specifically designed for the assessment of nutrition-related apps such as the App Quality Evaluation may provide more accurate results [56].

### Conclusions

This study provides a comprehensive overview of the most demanded nutrition-related apps in the Spanish app market. We found that the majority of the apps were appealing due to their user-friendly interfaces, potentially attracting a larger user base and enhancing adherence. However, few apps provided dietary structure analysis or nutritional education, hindering users’ ability to follow a well-balanced diet. Additionally, much of the information provided within the apps raises concerns about its validity. To ensure evidence-based content, collaboration between app developers and nutrition experts is crucial during the app’s design phase. Multidisciplinary health care teams, including dietitians, should support patients through mHealth apps to enhance patients’ long-term dietary lifestyle changes. Moreover, the findings of this study can help users choose suitable apps and support app developers in the development or refinement of nutrition-related apps. Further research is needed to assess the long-term impact of these apps on users.

## Supplementary material

10.2196/52424 Multimedia Appendix 1 The user version of the Mobile App Rating Scale total and section-specific scores of the nutrition-related apps (N=42).

10.2196/52424 Multimedia Appendix 2 Results of the total and section-specific quality assessment scores of the user version of the Mobile App Rating Scale assessment for the nutrition-related apps in this study (N=42).

## Abbreviations

MARS: Mobile App Rating Scale
mHealth: mobile health
PRISMA: Preferred Reporting Items for Systematic Reviews and Meta-Analyses
uMARS: user version of the Mobile App Rating Scale
