# Supplementary material for: Nutrition-Related Mobile Apps in the Spanish App Stores: Quality and Content Analysis
Source: JMIR Mhealth Uhealth. 2024 Oct 4;12:e52424. doi: 10.2196/52424 (PMC11474592; doi:10.2196/52424)
Supplement: Multimedia Appendix 1 [file mhealth-v12-e52424-s001.docx]

| uMARS scores | | Mean | SD | Min | Max | Median | Q1 | Q3 |
| --- | --- | --- | --- | --- | --- | --- | --- | --- |
| **Total** | | 3.78 | 0.35 | 2.93 | 4.39 | 3.78 | 3.57 | 4.07 |
|  | Engagement | 3.51 | 0.46 | 2.61 | 4.40 | 3.48 | 3.18 | 3.74 |
|  | Functionality | 4.21 | 0.38 | 3.10 | 4.75 | 4.31 | 4.04 | 4.47 |
|  | Aesthetics | 3.94 | 0.54 | 2.42 | 4.75 | 3.95 | 3.75 | 4.45 |
|  | Information | 3.48 | 0.44 | 2.51 | 4.25 | 3.59 | 3.04 | 3.84 |
| **Subjective quality** | | 2.65 | 0.56 | 1.67 | 3.94 | 2.52 | 2.25 | 2.95 |
| **Perceived impact** | | 3.06 | 0.67 | 1.90 | 4.20 | 3.13 | 2.57 | 3.59 |
| **Total by aim** | |  |  |  |  |  |  |  |
|  | Nutritional plans or diet | 3.87 | 0.33 | 3.40 | 4.26 | 3.85 | 3.63 | 4.10 |
|  | Recipes | 3.65 | 0.18 | 3.30 | 3.79 | 3.70 | 3.63 | 3.77 |
|  | Recording and analysis of food | 3.79 | 0.39 | 2.93 | 4.39 | 3.79 | 3.54 | 4.10 |
|  | Advice on healthy habits | 3.78 | 0.37 | 3.11 | 4.18 | 3.93 | 3.56 | 4.12 |

Abbreviations: max, maximum; min, minimum; Q1, quartile first; Q3, quartile third; SD, standard deviation; uMARS, user version of the Mobile App Rating Scale.
