# Supplementary material for: Nutrition-Related Mobile Apps in the Spanish App Stores: Quality and Content Analysis
Source: JMIR Mhealth Uhealth. 2024 Oct 4;12:e52424. doi: 10.2196/52424 (PMC11474592; doi:10.2196/52424)
Supplement: Multimedia Appendix 2 [file mhealth-v12-e52424-s002.docx]

|  | Reviewer 1 | | | | | | |  | Reviewer 2 | | | | | | |
| --- | --- | --- | --- | --- | --- | --- | --- | --- | --- | --- | --- | --- | --- | --- | --- |
| App | Total | Engagement | Functionality | Aesthetics | Information | Subjective Quality | Perceived Impact |  | Total | Engagement | Functionality | Aesthetics | Information | Subjective Quality | Perceived Impact |
| 1 | 3.96 | 3.78 | 4.28 | 4.19 | 3.60 | 3.15 | 3.74 |  | 3.71 | 3.30 | 4.68 | 3.30 | 3.58 | 2.48 | 2.75 |
| 2 | 3.53 | 3.42 | 4.28 | 3.29 | 3.15 | 2.25 | 2.70 |  | 2.32 | 2.42 | 2.75 | 2.20 | 1.93 | 1.10 | 1.10 |
| 3 | 3.93 | 3.78 | 4.50 | 3.60 | 3.83 | 3.15 | 3.90 |  | 2.81 | 1.98 | 3.03 | 4.03 | 2.20 | 1.10 | 1.10 |
| 4 | 3.99 | 3.96 | 4.50 | 3.90 | 3.60 | 3.60 | 3.74 |  | 2.99 | 3.52 | 3.30 | 2.93 | 2.20 | 1.38 | 1.83 |
| 5 | 3.89 | 3.78 | 4.28 | 3.90 | 3.60 | 2.93 | 2.55 |  | 4.25 | 3.52 | 4.68 | 4.67 | 4.13 | 3.58 | 4.03 |
| 6 | 4.14 | 3.96 | 4.28 | 4.50 | 3.83 | 2.70 | 3.45 |  | 3.21 | 3.30 | 2.75 | 4.03 | 2.75 | 1.65 | 2.57 |
| 7 | 3.97 | 3.60 | 4.28 | 4.19 | 3.83 | 3.38 | 3.29 |  | 3.28 | 2.86 | 4.40 | 3.67 | 2.20 | 2.20 | 2.38 |
| 8 | 3.69 | 3.60 | 4.28 | 3.29 | 3.60 | 2.70 | 2.39 |  | 3.35 | 2.86 | 3.58 | 4.77 | 2.20 | 1.93 | 2.02 |
| 9 | 4.12 | 3.96 | 4.50 | 4.19 | 3.83 | 3.60 | 4.19 |  | 4.08 | 4.40 | 4.68 | 3.67 | 3.58 | 3.03 | 3.85 |
| 10 | 3.88 | 3.24 | 4.50 | 4.19 | 3.60 | 2.70 | 2.10 |  | 2.72 | 1.98 | 3.85 | 2.57 | 2.48 | 1.93 | 2.02 |
| 11 | 4.30 | 4.14 | 4.50 | 4.50 | 4.05 | 3.83 | 4.05 |  | 4.35 | 4.40 | 4.75 | 4.67 | 3.58 | 3.03 | 4.03 |
| 12 | 4.07 | 3.78 | 4.50 | 4.19 | 3.83 | 3.15 | 3.45 |  | 4.53 | 4.60 | 4.13 | 5.00 | 4.40 | 3.58 | 4.22 |
| 13 | 3.70 | 3.42 | 4.50 | 3.29 | 3.60 | 2.48 | 2.84 |  | 4.19 | 3.74 | 4.40 | 4.77 | 3.85 | 1.93 | 3.67 |
| 14 | 4.06 | 3.96 | 4.50 | 4.19 | 3.60 | 3.15 | 3.74 |  | 4.44 | 3.96 | 4.68 | 5.00 | 4.13 | 2.75 | 4.22 |
| 15 | 4.14 | 3.96 | 4.50 | 4.50 | 3.60 | 3.15 | 3.74 |  | 3.50 | 2.64 | 4.50 | 4.67 | 2.20 | 1.38 | 2.75 |
| 16 | 3.15 | 3.06 | 2.93 | 3.00 | 3.60 | 2.03 | 2.10 |  | 3.18 | 3.08 | 3.85 | 3.30 | 2.48 | 1.93 | 3.12 |
| 17 | 3.96 | 3.78 | 4.28 | 4.19 | 3.60 | 3.15 | 3.74 |  | 4.10 | 4.84 | 3.85 | 3.30 | 4.40 | 3.85 | 4.50 |
| 18 | 3.68 | 3.24 | 4.50 | 3.60 | 3.38 | 2.03 | 1.94 |  | 3.13 | 3.08 | 4.13 | 2.57 | 2.75 | 2.48 | 2.38 |
| 19 | 3.42 | 3.42 | 4.28 | 3.00 | 3.00 | 2.70 | 2.10 |  | 4.08 | 3.96 | 4.40 | 3.30 | 4.68 | 4.13 | 4.83 |
| 20 | 3.93 | 3.42 | 4.50 | 4.19 | 3.60 | 2.93 | 3.90 |  | 3.77 | 4.18 | 3.85 | 4.03 | 3.03 | 2.48 | 3.48 |
| 21 | 3.78 | 3.42 | 4.50 | 3.60 | 3.60 | 2.70 | 2.55 |  | 3.67 | 3.96 | 3.58 | 3.30 | 3.85 | 1.93 | 4.58 |
| 22 | 3.72 | 3.42 | 4.28 | 3.60 | 3.60 | 2.70 | 2.70 |  | 3.54 | 3.08 | 4.13 | 3.67 | 3.30 | 2.75 | 3.12 |
| 23 | 4.12 | 3.96 | 4.50 | 4.19 | 3.83 | 3.15 | 3.29 |  | 4.21 | 4.18 | 4.13 | 4.40 | 4.13 | 3.58 | 4.03 |
| 24 | 4.16 | 4.14 | 4.50 | 4.19 | 3.83 | 3.38 | 3.90 |  | 4.61 | 4.18 | 5.00 | 4.77 | 4.50 | 4.50 | 4.50 |
| 25 | 4.08 | 3.96 | 4.50 | 4.50 | 3.38 | 3.38 | 2.84 |  | 3.15 | 2.42 | 4.13 | 4.40 | 1.65 | 1.10 | 1.10 |
| 26 | 3.89 | 3.78 | 4.05 | 3.90 | 3.83 | 3.60 | 3.15 |  | 4.56 | 4.84 | 4.68 | 4.03 | 4.68 | 3.58 | 4.58 |
| 27 | 3.43 | 3.24 | 3.60 | 3.29 | 3.60 | 2.48 | 2.25 |  | 3.40 | 2.86 | 3.85 | 3.30 | 3.58 | 1.38 | 3.30 |
| 28 | 3.77 | 3.24 | 4.28 | 4.19 | 3.38 | 2.25 | 2.00 |  | 4.59 | 4.18 | 5.00 | 4.77 | 4.40 | 2.75 | 4.03 |
| 29 | 3.71 | 3.06 | 4.28 | 4.19 | 3.29 | 2.03 | 1.83 |  | 3.87 | 3.30 | 4.40 | 3.67 | 4.13 | 3.58 | 3.48 |
| 30 | 3.47 | 2.70 | 4.28 | 3.60 | 3.29 | 2.00 | 1.50 |  | 4.06 | 3.08 | 5.00 | 4.03 | 4.13 | 3.58 | 4.58 |
| 31 | 4.15 | 3.78 | 4.50 | 4.50 | 3.83 | 3.38 | 2.84 |  | 2.98 | 2.86 | 3.58 | 3.30 | 2.20 | 1.65 | 1.10 |
| 32 | 3.87 | 3.42 | 4.28 | 4.19 | 3.60 | 2.48 | 1.66 |  | 3.67 | 3.96 | 3.58 | 3.30 | 3.85 | 1.93 | 4.58 |
| 33 | 4.01 | 3.96 | 4.28 | 4.19 | 3.60 | 3.15 | 3.15 |  | 3.22 | 3.52 | 3.58 | 3.30 | 2.48 | 1.93 | 3.12 |
| 34 | 3.83 | 3.24 | 4.50 | 4.19 | 3.38 | 2.25 | 1.94 |  | 4.41 | 3.74 | 5.00 | 4.77 | 4.13 | 2.75 | 4.40 |
| 35 | 3.87 | 3.24 | 4.50 | 3.90 | 3.83 | 2.25 | 1.83 |  | 3.42 | 2.86 | 3.85 | 3.67 | 3.30 | 1.93 | 3.30 |
| 36 | 3.48 | 3.06 | 4.28 | 3.00 | 3.60 | 1.75 | 1.83 |  | 2.74 | 3.08 | 1.93 | 1.83 | 4.13 | 3.03 | 4.50 |
| 37 | 4.08 | 3.96 | 4.05 | 4.50 | 3.83 | 3.38 | 3.29 |  | 4.43 | 4.84 | 5.00 | 4.03 | 3.85 | 4.13 | 4.77 |
| 38 | 3.93 | 3.42 | 4.50 | 4.19 | 3.60 | 2.70 | 3.15 |  | 3.91 | 3.08 | 4.50 | 4.77 | 3.30 | 2.75 | 4.03 |
| 39 | 3.87 | 3.42 | 4.28 | 4.19 | 3.60 | 3.15 | 2.10 |  | 3.83 | 3.52 | 4.40 | 4.67 | 2.75 | 2.20 | 2.57 |
| 40 | 3.89 | 3.42 | 4.50 | 3.60 | 4.05 | 2.03 | 2.84 |  | 3.23 | 2.20 | 3.85 | 4.40 | 2.48 | 1.38 | 2.02 |
| 41 | 4.08 | 3.96 | 4.28 | 4.50 | 3.60 | 2.48 | 2.55 |  | 3.81 | 2.86 | 4.13 | 4.40 | 3.85 | 2.75 | 3.85 |
| 42 | 3.85 | 3.24 | 4.28 | 4.50 | 3.38 | 2.03 | 1.33 |  | 4.01 | 3.52 | 4.50 | 5.00 | 3.03 | 2.48 | 2.75 |
